# Endothelial Tumours

**Published:** 1907-12-21

**Authors:** 


### Points in Pathology.

### ENDOTHELIAL TUMOURS.

Of late years much attention has been given to the class of tumours known as the endotheliomata—that is to say, tumours arising from the endothelial cells covering the surface of the peritoneum, pleura, and cerebro-spinal membranes, or lining the small vascular and lymphatic channels. That the peritoneum, the dura mater, and other serous membranes may be the seat of new growths of an endothelial nature has been recognised for some time, and it has also been urged, especially by certain German pathologists, that the curious " mixed " tumours of the parotid and submaxillary glands are, in part at least, of a similar kind. But it seems probable that our conception of the endotheliomata must be extended so as to include, in addition to these examples, certain tumours found in other parts of the body, particularly in the breast and the cervix uteri; tumours very nearly resembling the glandular cancers, for which they are often mistaken.

Assuming that the vascular and lymphatic endothelium is capable of proliferation, this proliferation may, on purely hypothetical grounds, occur in three different ways. It may occur centrifugally, in the sense of being directed away from the lumen of the vessel; it may be centripetal, the proliferated cells filling up and obliterating the lumen of the vessel; it may be both centrifugal and centripetal. In the first case the microscope will show a collection of cells arranged round a small vessel, from whose walls the cell-growth originates; in the second case we shall see a mass of cells lying in what was once a lymph or blood channel; in the third case the arrangement will be very similar to that in the second, except that the cell-mass will be larger. Moreover, each of these varieties of growth will constitute a hæmal or a lymphatic endothelioma, according as it originates from a blood or a lymph channel.

Let us now consider how these endothelial tumours come to be confused with the carcinomata. Although the normal endothelial cells lining a vessel are sufficiently characteristic in appearance, it is almost impossible to distinguish them, when proliferated, from the spheroidal cells of a glandular cancer. In either case the cells are roughly circular in shape, or polygonal from mutual pressure, each containing a well-defined vesicular nucleus; and as there is, at present, no stain which can differentiate between an endothelial and an epithelial cell, any differentiation must in this case depend on tracing the origin of the cell either from a gland tubule or from an endothelial membrane. Now when the development of an endothelioma has taken a centrifugal direction, the arrangement of cells radiating from a central space immediately suggests that the growth is a carcinoma, and that the central space is a glandular acinus, especially in the breast or the uterus, where carcinoma is so often found. In the case of a hæmal endothelioma of this nature a fortunate specimen may show some blood-corpuscles lying in the spaces, and this will at once prove these spaces to be blood-vessels, and not gland tubules. But in the case of a lymphatic endothelioma this criterion will not be available, for the spaces will contain nothing but a little coagulated lymph. To determine the nature of such a tumour we must, therefore, examine its growing edge and see that these channels are lymphatic and not glandular. When the proliferation of the endothelium has occurred centripetally a correct diagnosis is even more difficult, for here we see merely a solid mass of cells containing no spaces, and it is again necessary to trace the origin of these cells at the margin of the growth before they can be identified as endothelial.

The clinical features of the endotheliomata are varied and inconstant. Some have no malignant attributes, as, for instance, the psammoma of the cerebral membranes, which neither infiltrates surrounding structures nor produces metastatic deposits, and causes symptoms only by reason of the pressure it exerts on the brain. Others, again, are typically malignant in that they rapidly infiltrate the neighbouring tissues and cause widespread metastases in the lymphatic glands and other organs; under this heading fall the endothelial tumours of the peritoneum, the breast, and the uterus. Midway between these two extremes stand the " mixed " tumours of the salivary glands. These form attachments to the sterno-mastoid muscle, the carotid sheath, and the cranial bones; they often implicate the facial nerve, and may produce ulceration of the skin over them, but they rarely form secondary deposits. On occasion, however, these tumours may depart from the usual sequence of events, spreading in all directions, both locally and in the lymphatic circulation; or, again, they may remain circumscribed for many years, and then suddenly take on all the features of malignancy. This inconsistency corresponds, perhaps, to their varying composition; those containing a larger proportion of cellular elements growing more rapidly than those consisting chiefly of cartilage and myxomatous material. It is also, probably, incorrect to class all tumours of the salivary glands in one group.

The question of the nature and origin of the endotheliomata is, for two reasons, of more than academic interest. In the first place, there is a growing idea that the sarcomata may eventually be proved to differ from the carcinomata as regards their ætiology, that they may perhaps be of an infective nature and fall into line with the infective granulomata, with certain of which they have many points in common. On account of their origin from a mesoblastic structure the endothelial tumours must be classed with the sarcomata rather than the carcinomata, although as regards their cellular constitution they bear an anomalous resemblance to the latter; they will, therefore, be included in this infective hypothesis. In the next place, it is possible that the endothelial tumours of the breast and uterus will be found to possess an even more pronounced malignancy than the carcinomata. Sufficient evidence on this point is not yet to hand, but the examination of a number of cases seems to point to the truth of this suggestion.

